# Hepatic Vessel Map (HVM): An Expert-Annotated CT Dataset for Clinically Applicable AI in Liver Vascular Segmentation and Surgical Planning

**DOI:** 10.1038/s41597-026-07550-3

**Published:** 2026-06-02

**Authors:** Tingting Xie, Xunqi Li, Linyu Zhang, Xuankai Huang, Ziwei Liu, Chunlan Huang, Qi Cai, Zixin Zhang, Chao Wang, Xilun Ma, Ruibin Huang, Zhendong Luo, Guanxun Cheng, Demin Xu, Zaiyi Liu, Cheng Lu

**Affiliations:** 1 Guangdong Cardiovascular Institute, Guangdong Provincial People’s Hospital, Guangdong Academy of Sciences, Guangzhou, 510080 China; 2 Department of Radiology, Guangdong Provincial People’s Hospital, Guangdong Academy of Medical Sciences, Southern Medical University, Guangzhou, 510080 China; 3 Guangdong Provincial Key Laboratory of Artificial Intelligence in Medical Image Analysis and Application, Guangzhou, 510080 China; 4 Medical imaging center, Peking University Shenzhen Hospital, Shenzhen, 518036 China; 5 Computer and Information Security, Guilin University of Electronic Technology, Guilin, 541004 China; 6 Medical Research Institute, Guangdong Provincial People’s Hospital, Guangdong Academy of Medical Sciences, Southern Medical University, Guangzhou, 510080 China; 7 Departments of Radiology, Guangxi Medical University Cancer Hospital, Nanning, 530012 China; 8 Department of Radiology, The First Affiliated Hospital of Shantou University Medical College, Shantou, 515041 China; 9 Department of Radiology, The University of Hong Kong - Shenzhen Hospital, Shenzhen, China

## Abstract

Precise delineation of hepatic and portal venous anatomy is crucial for the diagnosis of liver disease, surgical planning, and prognosis prediction. Current three-dimensional visualization of these complex vascular structures relies on manual or semi-automated CT segmentation, which is time-consuming and operator-dependent. Although artificial intelligence (AI) presents a promising alternative, existing methods remain constrained by the scarcity of publicly available datasets with fine-grained vascular annotations and inadequate validation in real-world diseased liver populations, which represent the majority of patients undergoing hepatic procedures. To address this gap, we present the Hepatic Vessel Map (HVM) Dataset, a dual-center resource comprising contrast-enhanced CT scans from 282 patients with over 4,1400 slices and 4,8300 annotations, each with meticulously annotated hepatic veins, portal veins (to third-order branches), and liver tumors. The dataset comprises a substantial proportion of cases with underlying hepatic pathology and has been validated for use in preoperative planning for major hepatectomy, ensuring both clinical relevance and model generalizability. This dataset supports: 1) development and benchmarking of robust hepatic and portal venous segmentation models; 2) vasoimcs research through quantitative analysis of vascular morphology, topology, and radiomic features; 3) generation of patient-specific 3D “digital vascular roadmaps” to enhance surgical precision and safety. As such, this dataset establishes a foundational resource for advancing AI-driven innovations in hepatobiliary surgery and intervention.

## Background & Summary

Accurate delineation of hepatic and portal venous anatomy is critical for the diagnosis of liver disease^1,2^, surgical planning^3^, and prognosis prediction^4^. These vessels serve as anatomical landmarks and a surgical “navigation map”^5^ in procedures such as living donor liver transplantation^6^, anatomical liver resection^7^, transjugular intrahepatic portosystemic shunt (TIPS)^8^, and portal vein embolization (PVE)^6,9^. The success of these procedures relies heavily on detailed preoperative visualization of the double venous liver system, including its overall configuration, the precise course of minor branches, and the spatial relationship between the tumors and adjacent venous structures.

Contrast-enhanced CT is the most widely used imaging modality for preoperative planning, providing high-resolution anatomy that enables surgeons to anticipate vascular variations, define optimal resection planes to avoid major vascular injury, and maximize the preservation of functional liver volume— thereby reducing the risk of lift-threatening postoperative complications^10–12^. However, the current clinical workflow for 3D vascular reconstruction remains predominantly manual or semi-automated, relying on slice-by-slice vessel segmentation at post-processing workstations^10,13^. This approach is not only labor-intensive and time-consuming but also operator-dependent, limiting its efficient application in routine clinical practice.

Although several AI-based segmentation models have been developed for automatic delineation of hepatic veins and portal veins^2,14–16^, significant limitations remain. First, existing AI models typically focus only on the main trunks and first-order branch ramifications, often failing to capture fine structural details of minor branches^2^ or to accurately differentiate between hepatic veins and portal veins^10,16^—a shortcoming that may lead to incorrect identification of the resection planes in liver surgery. Second, there is a notable lack of validation data derived from diseased livers in real-world preoperative settings^14,15^, raising concerns regarding model generalizability. Since most patients undergoing liver surgery present with underlying hepatic pathology, such pathological alterations— including reduced hepatic vascular volume, increased venous tortuosity, portal vein embolism, and alterations in venous morphology and topology caused by tumor invasion or compression— can significantly impair the segmentation accuracy of these AI models in clinical scenarios^2,17^.

The development of clinically applicable AI models critically depends on high-quality liver vascular databases. However, current publicly available datasets suffer from the following limitations that potentially hinder the development of such AI models:

1. **Insufficient or incomplete vascular annotations:** Most datasets, such as LiTS^18^ and SLiver07^19^, focus exclusively on liver parenchyma and tumor segmentation, providing no vascular labels. The LiVS^20^ dataset, one of the few publicly available resources that includes vessel annotations, is limited to annotating only 30 random two-dimensional slices per scan and lacks hepatic veins labels. This sparse and inherently discontinuous sampling method is inadequate for training models that require an understanding of complete vascular topology—an essential prerequisite for precise surgical planning and navigation.
2. **Limited generalizability to diseased livers:** Most existing vessel segmentation models have been developed and validated on datasets derived predominantly from healthy livers or populations with minimal pathology^20,21^. This raises concerns about their generalizability to the diseased livers that constitute the majority of surgical candidates, where pathological alterations—such as reduced vascular volume, increased venous tortuosity, portal vein embolism, and morphological changes caused by tumor invasion or compression—can significantly impair segmentation accuracy.

Our dataset addresses the above-mentioned unmet needs. We present a dual-institutional, contrast-enhanced CT dataset specifically designed to support the development of precise and clinically applicable segmentation models for hepatic veins and portal veins, thereby contributing to vascular AI research and the advancement of next-generation surgical planning systems. The core value of this dataset lies in three key contributions:

1. **Fine-Grained, Continuous Expert Annotations for Clinically Relevant Vascular Segmentation**: In contrast to the sparse and incomplete annotations in existing datasets, our dataset provides continuous, full-volume, fine-grained annotations of both hepatic and portal venous systems down to the second-to-fourth order branches, along with detailed liver tumor delineations. This design captures fine vascular details and supports accurate differentiation between hepatic and portal veins, establishing a robust foundation for training clinically applicable segmentation models.
2. **Real-World Clinical Validation and Generalizability**: Unlike prior datasets that predominantly include healthy livers, over half of the cases (59.57%) in our dataset are derived from patients with underlying hepatic pathology (e.g., fatty liver, cirrhosis). Importantly, more than half (62.41%, cases in Center 2) of the included cases have been previously used for preoperative assessment of hepatic veins, portal veins, and future liver remnant volume before major hepatectomy. This real-world clinical grounding ensures that models trained on our dataset are exposed to the anatomical variability and pathological changes commonly encountered in surgical practice, thereby enhancing their potential for generalization to real clinical settings.
3. **Enabling AI-Driven Surgical Navigation and Decision Support**: By integrating continuous, fine-grained vascular annotations with tumor masks and real-world clinical data, this dataset supports the development of segmentation models that can generate patient-specific 3D “digital vascular roadmaps.” Such roadmaps support preoperative planning, surgical simulation, and prediction of postoperative liver function—which is linked to post-hepatectomy liver failure—thereby enhancing procedural safety and planning accuracy.

**Fig. 1:**
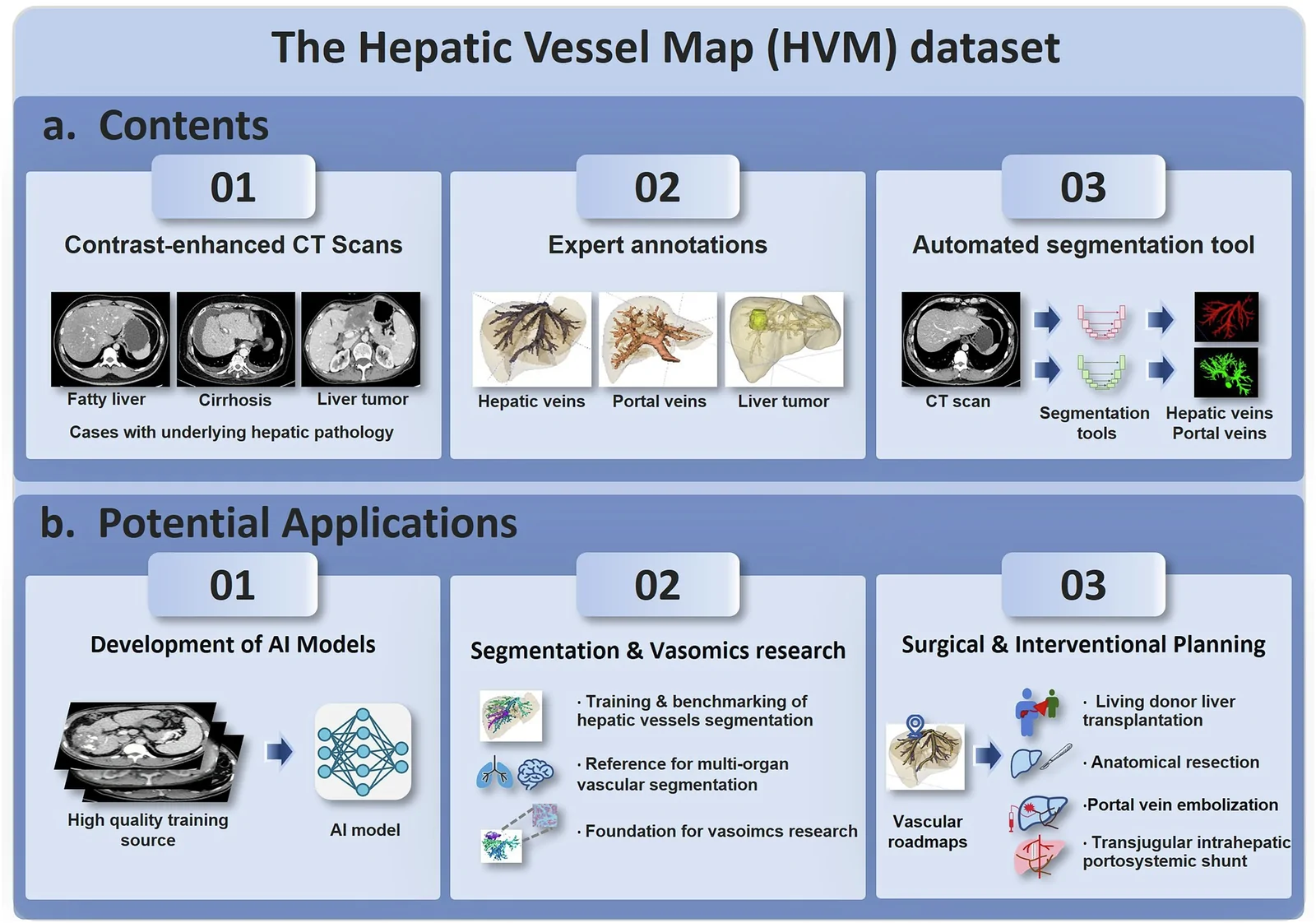
Overview and potential applications of the hepatic vessel map (HVM) dataset.

By integrating high-quality imaging and meticulously expert-annotated segmentation of the hepatic veins and portal veins (as demonstrated in Fig. 1a), this dataset provides a foundational resource for developing robust and generalizable segmentation models of hepatic veins and portal veins. Potential applications of this dataset included the development of AI models, segmentation and vasomics research, and support for hepatic surgical and interventional planning- such as living donor liver transplantation, anatomical hepatectomy, PVE, and TIPS (Fig. 1b). This dataset serves as an indispensable resource for advancing both AI-driven research and downstream clinical practice in hepatobiliary surgery and intervention procedures.

## Methods

### Ethical statement

This retrospective study was approved by the Ethics Committee of Peking University Shenzhen Hospital [IRB numbers: 2021(071) and 2021(071)-1] and the Ethics Committee of University of Hong Kong - Shenzhen Hospital [IRB numbers: 2025 (266)]. Given the use of anonymized patient data throughout the study and the retrospective nature, the requirement for informed consent was waived by the ethical standards outlined in the Declaration of Helsinki and its later amendments.

### Patient cohorts

**Fig. 2:**
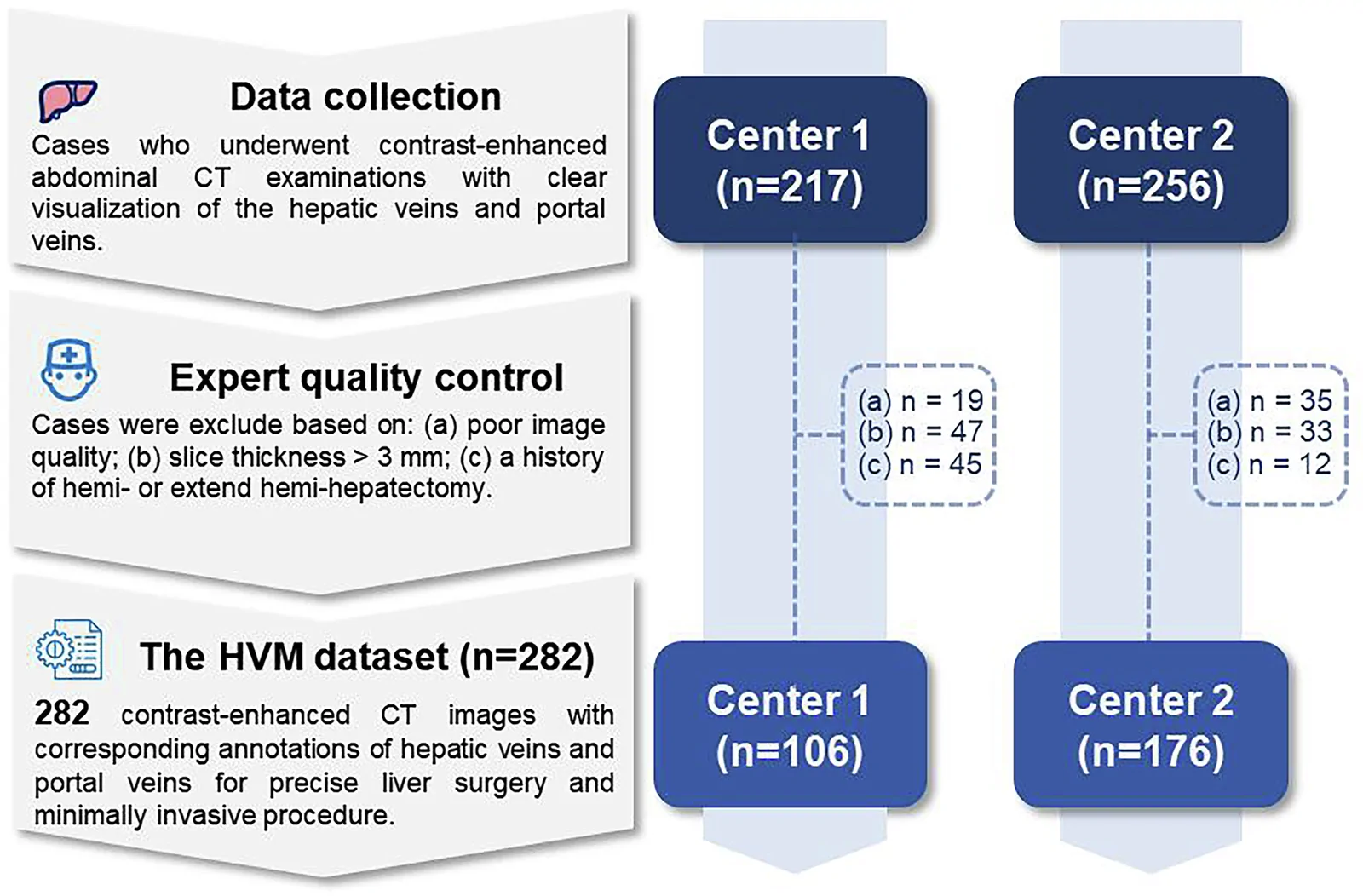
Flowchart of building the HVM dataset.

Consecutive patients who underwent contrast-enhanced abdominal CT examinations were retrospectively collected from Center 1 (The University of Hong Kong - Shenzhen Hospital) between June 2019 and December 2021, and from Center 2 (Peking University Shenzhen Hospital) between January 2018 and March 2019. The final dataset included 282 individuals, with 106 from Center 1 and 176 from Center 2. Thirty-two patients who underwent liver resection for liver tumors and were confirmed by histopathological results were collected in Center 2. The other liver tumors in both cohorts were verified based on radiological diagnostic reports. The CT images in Center 2 were previously used for the development of segmentation models for hepatic veins and portal veins^22^. A detailed patient inclusion flowchart is presented in Fig. 2.

Inclusion criteria were as follows: (a) Age ≥ 18 years; (b) Availability of portal venous phase or delay phase contrast-enhanced CT images with clear visualization of hepatic veins and portal veins. The following cases were excluded: (a) poor image quality due to obvious artifacts affecting vascular interpretation; (b) slice thickness exceeding 3 mm; (c) history of hemi-hepatectomy or extended hemi-hepatectomy.

### Clinical characteristics

Clinical variables were collected, comprising age and gender for the entire cohort. The average volume of liver tumors was calculated based on measurements derived from contrast-enhanced CT imaging. For the subset of patients who underwent liver resection, pathological diagnosis confirmed by histopathology was recorded. The pathological diagnosis in Center 1 was not available in Center 1, as these patients did not undergo liver resection or liver biopsy. The liver condition of non-tumor tissue was confirmed based on the radiological diagnostic report, as summarized in Table 1.

**Table 1:** Clinical characteristics of patient data.

Characteristics	Center 1 (n = 106)	Center 2 (n = 176)
Age (years)	58.71 ± 11.15	48.75 ± 15.40
Gender (%)		
Male	67(63.21)	108(61.36)
Female	39(36.79)	68(38.64)
Average volume of liver tumors (cm^3^)	2.02 ± 4.13	83.63 ± 60.68
Pathological result (%)		
Hepatocellular carcinoma	NA	27(15.34)
Intrahepatic cholangiocarcinoma	NA	1(0.57)
Hepatic metastasis	NA	1(0.57)
Hepatic hemangioma	NA	3(1.70)
Liver condition of non-tumor tissue		
Reported healthy liver	41(38.68)	73(41.48)
Steatosis	30(28.30)	50(28.41)
Cirrhosis	35(33.02)	53(30.11)

-Note. NA: not available.

### CT image acquisition

All CT examinations were performed using multi-detector CT scanners from three different manufacturers. A standardized three-phase contrast-enhanced protocol was used. Iodinated contrast agent (1.5 mL/kg body weight) was administered intravenously at a rate of 3–4 mL/s. The late arterial phase scans were triggered using bolus tracking, with scan initiation set at 25–30 seconds post-injection. The portal venous phase was acquired 60–70 seconds after contrast administration. CT scanning parameters were summarized in Table 2. The involvement of multiple scanners and manufacturers introduces inherent variability, enhancing the dataset’s robustness and generalizability.

**Table 2:** CT Scanning parameters across centers.

Category	Center 1(n = 106)	Center 2(n = 176)
CT scanner (n, %)		
GE Light Speed VCT	25(23.58)	0(0)
GE Discovery CT750 HD	17(16.04)	0(0)
GE Revolution	0(0)	107(60.80)
Philips Brilliance iCT 256	48(45.28)	0(0)
Siemens Definition Flash	16(15.09)	69(39.20)
Tube voltage (n, %)		
120 kVp	170(100)	133(75.57)
100 kVp	0(0)	43(24.43)
Slice thickness (mm)		
1.0	64(60.37)	69(39.20)
1.25	42(39.63)	107(60.80)

### Expert annotations and segmentation protocol

The manual segmentation of hepatic veins, portal veins, and liver tumors was performed on the portal venous phase images, where vascular contrast opacification is optimal. All segmentations were carried out using ITK-SNAP software (version 3.8.0, www.itksnap.org).

A rigorous two-tier annotation protocol was employed to ensure the quality of the ground-truth labels:

1. **Initial Segmentation:** A radiologist with 5 years of experience in abdominal imaging performed the initial manual segmentation for all cases.
2. **Quality Control and Refinement:** A senior abdominal radiologist with more than 15 years of expertise reviewed all initial segmentations, corrected any inaccuracies, and provided final validation, thereby establishing the consensus-based ground truth for the dataset.

**Fig. 3:**
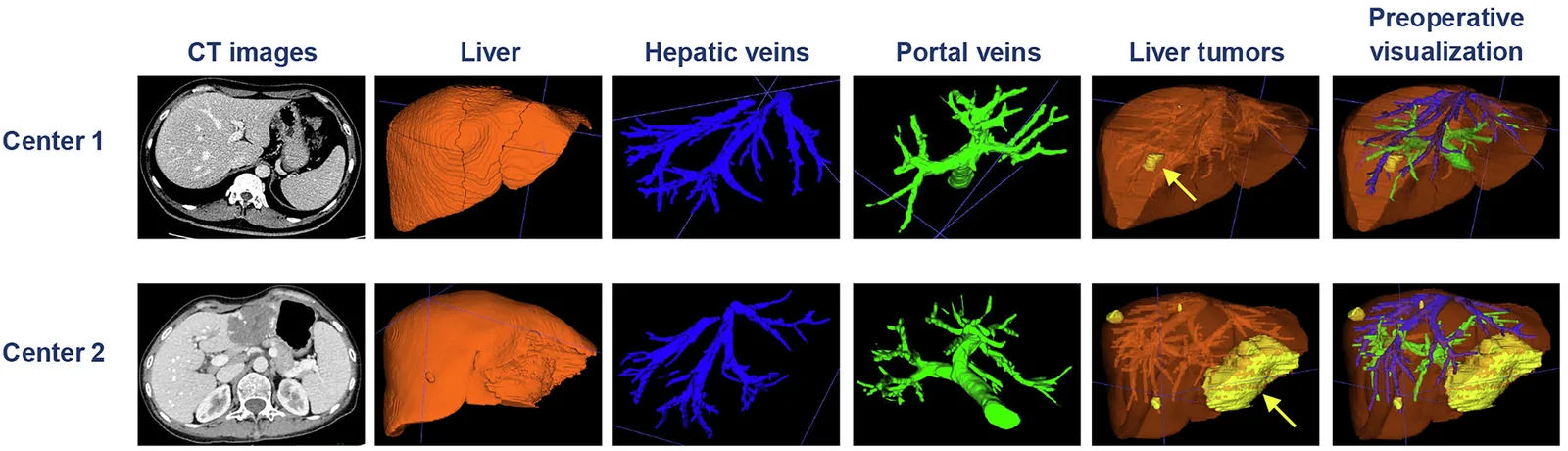
Examples of portal venous phase (PVP) contrast-enhanced CT images and corresponding annotations. The first column shows PVP CT images obtained from the two centers. The second to the fifth columns illustrate the annotations of the hepatic veins, portal veins, liver tumor (indicated by arrows), and the preoperative visualization of the spatial relationship between liver tumors and intrahepatic vascular, respectively.

The annotation of the hepatic veins encompassed the right, middle, and left hepatic veins, as well as all visible tributaries up to the third-order branches. For the annotation of portal veins, the main portal vein was fully annotated, and the left portal vein and right portal vein were annotated up to the third branch of the ramification. Examples of the original CT images and the corresponding expert annotations are demonstrated in Fig. 3.

### Data preprocessing and De-identification

All source Digital Imaging and Communications in Medicine (DICOM) files were processed to ensure patient privacy and data consistency. Personally identifiable information was thoroughly removed in compliance with HIPAA regulations. The DICOM files were then converted into the Neuroimaging Informatics Technology Initiative (NIfTI) format (with.nii.gz extension) using the dcm2niix tool (version v1.0.20230411), which was configured to perform anonymization during the conversion.

## Data Records

The “HVM Dataset” (Hepatic Vessel Map Dataset) has been made publicly accessible via Zenodo^23^ (https://zenodo.org/records/19885789). Distributed under a CC-BY license, in accordance with its original ethics waiver for data assembly, this resource requires appropriate citation in any downstream use.

**Fig. 4:**
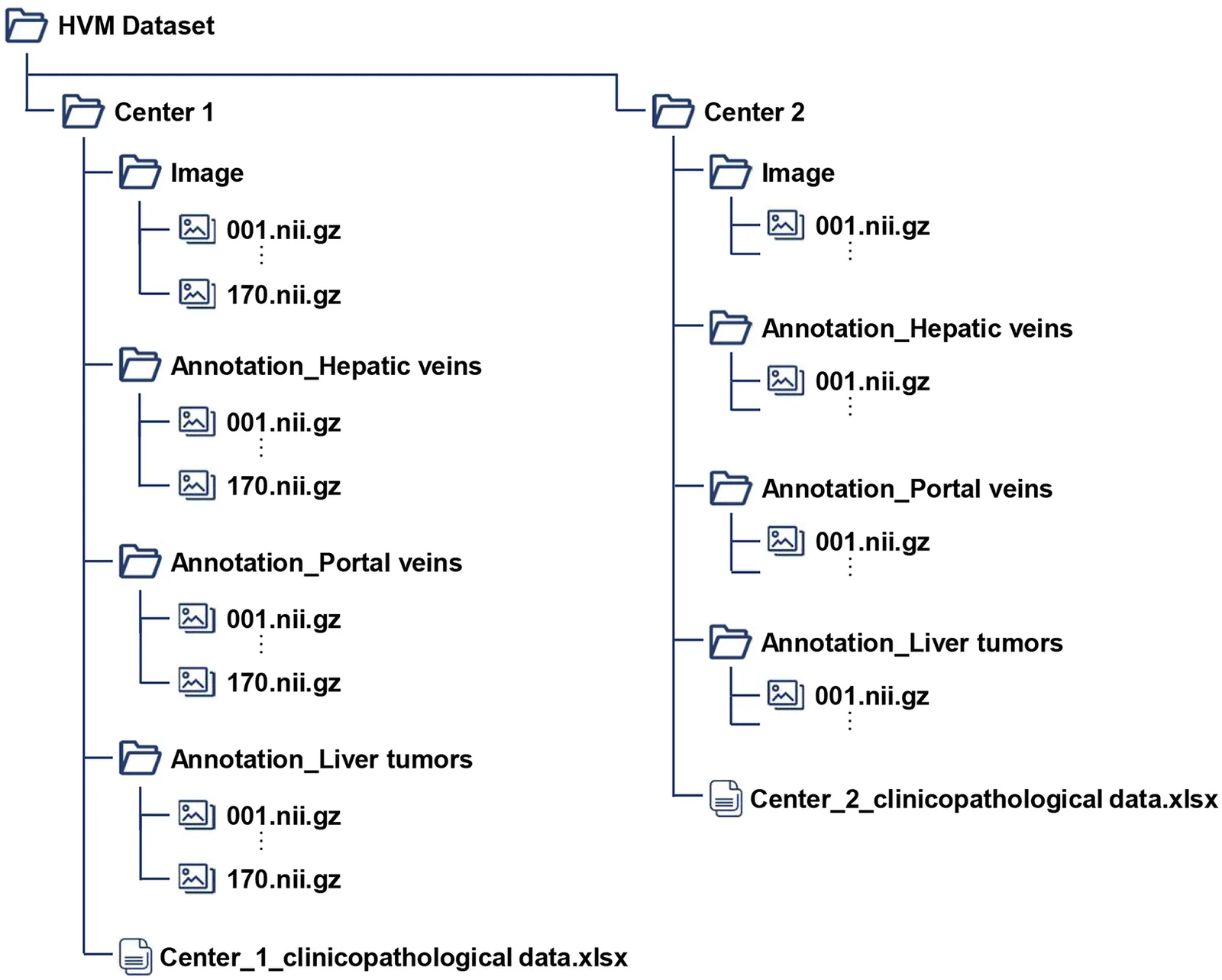
The Hepatic Vessel Map (HVM) dataset’s data structure, format, and nomenclature.

As outlined in Fig. 4, the dataset is structured with a main directory titled “HVM Dataset” which contains two subfolders—“Center 1” and “Center 2”—each storing the PVP contrast-enhanced CT images from the corresponding institution.

Within each center-specific folder, the data are organized into the following subdirectories:

1. An “Image” subfolder for the center’s PVP contrast-enhanced CT scans in Nifti format.
2. An “Annotation_Hepatic veins” subfolder for the segmentations of the hepatic venous.
3. An “Annotation_Portal veins” subfolder for the segmentations of the portal venous.
4. An “Annotation_Liver tumors” subfolder for the segmentation of liver tumors.
5. An Excel file (Center_X_Clinicopathological data.xlsx) that contains relevant clinicopathological variables, which can be linked to imaging data through a unique “Patient-ID” field.

Notably, the dataset includes only the images and manual segmentations, omitting any precomputed radiomic features. This intentional design affords researchers the flexibility to implement custom feature extraction pipelines tailored to their specific analytical goals.

## Technical Validation

### Quality control for images

Each CT scan underwent a two-stage quality assessment. First, subject eligibility was confirmed based on the predefined inclusion criteria. Second, a radiologist with over 15 years of clinical experience performed a visual inspection of each scan to confirm the absence of significant artifacts and to verify adequate vascular contrast opacification for subsequent segmentation.

### Annotation quality control

To ensure the quality of the annotations of hepatic veins and portal veins, a rigorous, multi-step protocol was implemented, combining manual expertise and AI-assisted refinement.

Step 1, establishment of initial ground truth: a junior radiologist (with an experience of 5 years) manually segmented the hepatic veins and portal veins in the first subset of 40 cases. These initial annotations were then reviewed, corrected where necessary, and finalized by a senior abdominal radiologist (with an experience of 15 years), establishing a high-quality ground truth set.

Step 2, AI model training: the ground truth set was used to train two dedicated 3D-UNet models for automated segmentation of the hepatic veins and portal veins.

Step 3, AI-assisted annotation and final validation: the trained models were applied to the remaining 242 cases to generate preliminary segmentation masks. To minimize potential bias introduced by AI-generated preliminary segmentations, the AI-predicted masks were displayed with high transparency, allowing radiologists to focus on the original CT appearance rather than the AI contours. The junior radiologist then manually corrected these AI-generated masks, and all corrections underwent a final review and approval by the senior radiologist to produce the expert consensus annotations. This two-tier expert review process ensures that the final labels reflect expert judgment rather than model bias.

For the annotations of liver tumors, a two-tiered manual annotation procedure was implemented. First, a radiologist with 5 years of experience outlined the boundaries of all liver tumors on PVP CT images. Subsequently, a senior abdominal radiologist with 15 years of expertise performed a comprehensive quality review and introduced further refinements as necessary.

The final consensus annotations of hepatic veins, portal veins, and liver tumors, serving as clinically validated ground truth labels, are included in this dataset.

### Inter- annotator consistency assessment

To quantitatively validate the reliability of manual annotations, we evaluated inter- annotator agreement between the two radiologists across both participating centers. The Dice similarity coefficient (DSC) was computed for each annotated structure—hepatic veins, portal veins, and liver tumors—using the complete set of annotations from all cases. As summarized in Table 3, the DSC values demonstrate consistently high agreement across all structures and centers, reflecting the reproducibility of our annotation protocol.

**Table 3:** Inter-annotator agreement (Dice similarity coefficient, DSC) for anatomical structure annotations.

DSC	Hepatic veins	Portal veins	Liver tumors
Center 1	0.91 ± 0.08	0.92 ± 0.07	0.94 ± 0.05
Center 2	0.90 ± 0.13	0.89 ± 0.09	0.92 ± 0.03

### Annotation time

The junior radiologist spent an average of 1.5 hours per case for manual correction of AI-generated masks, and the senior radiologist spent 0.5 hours per case for final review, reflecting substantial expert oversight.

## Comparison of our dataset with existing publicly available liver vessel segmentation benchmarks

Table 4 provides a systematic comparison between our HVM dataset and existing publicly available liver vessel segmentation benchmarks across three key dimensions: scale, annotation detail, and clinical relevance & validation depth.

**Table 4:** Comparison of the HVM dataset with existing publicly available liver vessel segmentation benchmarks.

Features	LiTS^18^	MSD^24^ Task08	LiVS^20^	HVM (Ours)
Sample size (patients)	201	443	532	282
Slice thickness (mm)	0.45–6	2.5–5	2.5–5	1–1.25
HV annotation	X	√, but HV and PV are not separated.	X	√, HV and PV are separated.
PV annotation	X	√, but HV and PV are not separated.	√	√, HV and PV are separated.
Vessel annotation detail	X	Full-volume, continuous, main + minor branches	30 random slices/scan, main + minor branches	Full-volume, continuous, main + minor branches
Liver tumor annotation	√	X	X	√
Diseased liver population	NA	NA	NA	Fatty livers/Cirrhosis
Clinical validation	X	√	X	√
Imaging modality	CT	CT	CT	CT

-Note. NA: not available; HV: hepatic veins; PV: portal veins.

In terms of scale, our dataset offers 282 patients, over 41,400 slices, and a slice thickness of 1–1.25 mm- a combination of high resolution and sufficient sample size that enables training of models capable of capturing fine vascular details. Regarding annotation detail, unlike Medical Segmentation Decathlon (MSD)^24^ challenge Task08 (which does not separate hepatic veins from portal veins) and LiVS^20^ (which provides only sparse, portal-vein-only annotations without hepatic veins), our dataset provides continuous, full-volume, separate annotations of hepatic and portal veins down to minor branches. This feature has the potential to support vasomics research, AI-driven vessel differentiation, and patient-specific surgical roadmaps. Critically, in terms of clinical relevance and validation depth, all scans are derived from real-world clinical practice and include pathological livers, with over 62% of cases previously validated in actual preoperative planning for major hepatectomy. This ensures generalizability to diseased livers encountered in routine surgery and facilitates the clinical translation of AI-based segmentation tools.

## Usage Notes

This contrast-enhanced CT dataset, featuring continuous expert annotations of the hepatic and portal venous system along with liver tumor annotations, serves as a valuable resource for AI research and clinical translation in liver surgery and image-guided interventions (as demonstrated in Fig. 1b). This dataset enables diverse downstream applications, organized into three primary domains:

1. **AI model development and validation**: Its primary application lies in the training, validating, and benchmarking state-of-the-art segmentation models for hepatic veins and portal veins. The inclusion of fine-grained annotations down to minor branches supports the development of models capable of distinguishing hepatic vein and portal veins and capturing anatomically relevant details. Additionally, the high-quality, multi-phase CT images provide a robust substrate for pre-training or fine-tuning abdominal-focused AI models in medical imaging.
2. **Vasomics research and cross-organ vascular segmentation:** Beyond direct segmentation tasks, the expert annotations establish a reliable basis for vasomics research^25^— the quantitative extraction of morphological, topological, and radiomic features from vascular structures. This enables research into imaging biomarkers related to liver function, portal hypertension, and tumor vascularity. Furthermore, the learned vascular features help to inform and accelerate the development of segmentation models for other organ systems with complex vasculature, such as the pulmonary and cerebral circulations.
3. **Clinical translation and surgical planning**: The integrated imaging and annotations allow for the generation of patient-specific 3D “digital vascular roadmaps,” which enhance preoperative planning and intraoperative guidance for a range of complex procedures. These include living donor liver transplantation, anatomic hepatectomy, PVE, and TIPS. By providing a realistic and annotated dataset derived from clinical practice and validated before major hepatectomy, this resource bridges technical AI innovation with tangible improvements in procedural safety, precision, and accessibility.

The dataset’s composition, which incorporates data from multiple scanners and a cohort encompassing both candidates of major hepatectomy with large liver masses and underlying liver pathology, as well as patients without liver tumors, is designed to enhance the robustness and generalizability of trained models. Furthermore, the dataset is structured in full compliance with the FAIR^26^ (Findable, Accessible, Interoperable, and Reusable) principles, thereby facilitating open science and reproducible research.

## Data Availability

The dataset described in this study is accessible at Zenodo^23^ (https://zenodo.org/records/ 19885789).

## Code availability

To facilitate immediate use, we provide a baseline nnU-Net model for the automated segmentation of hepatic veins and portal veins. The code, along with pre-trained weights and instructions for inference, is available on GitHub at: https://github.com/zhengyidong135/nnunet.
